# Tear proteomic profile in three distinct ocular surface diseases: keratoconus, pterygium, and dry eye related to graft-versus-host disease

**DOI:** 10.1186/s12014-020-09307-5

**Published:** 2020-12-07

**Authors:** Daniel de Almeida Borges, Marcos Rodrigo Alborghetti, Adriana Franco Paes Leme, Romenia Ramos Domingues, Bruna Duarte, Melina Veiga, Marilia Trindade Ferrer, Ana Claudia Viana Wanzeler, Carlos Eduardo Leite Arieta, Monica Alves

**Affiliations:** 1 Department of Ophthalmology and Otorhinolaryngology, Faculty of Medical Sciences, University of Campinas (UNICAMP), Campinas, São Paulo Brazil; 2 Department of Cell Biology, University of Brasilia, Brasilia, Brazil; 3 Brazilian Biosciences National Laboratory (LNBio), Brazilian Center for Research in Energy and Materials (CNPEM), Campinas, Brazil

**Keywords:** Keratoconus, Dry eye, Pterygium, Tear film, Proteomics

## Abstract

**Background:**

Diseases of the anterior segment of the eye may present different mechanisms, intensity of symptoms, and impact on the patients’ quality of life and vision. The tear film is in direct contact with the ocular surface and cornea and can be easily accessed for sample collection, figuring as a promising source of potential biomarkers for diagnosis and treatment control. This study aimed to evaluate tear proteomic profile in 3 distinct ocular diseases: keratoconus (corneal ectasia), severe dry eye related to graft-versus-host-disease (tear film dysfunction and ocular inflammatory condition) and pterygium (conjunctival fibrovascular degenerative disease).

**Methods:**

Tear samples were collected from patients of each condition and a control group. By using mass spectrometric analysis combined with statistics and bioinformatics tools, a detailed comparison of protein profile was performed.

**Results:**

After Student’s t-test analyses comparing each condition to the control group, we found the following number of differentially expressed proteins: 7 in keratoconus group, 29 in pterygium group, and 79 in GVHD group. Following multivariate analyses, we also report potential candidates as biomarkers for each disease.

**Conclusions:**

We demonstrated herein that mass spectrometry-based proteomics was able to indicate proteins that differentiate three distinct ocular conditions, which is a promising tool for the diagnosis of ocular diseases.

## Background

Ocular surface diseases encompass a wide range of conditions associated with corneal and conjunctival structures, tear film imbalance and adnexal glands dysfunction. Distinct disorders may commune similar clinical presentation despite significant differences in pathophysiological mechanisms [1]. Tear fluid plays an essential role in the ocular surface through its lubricating properties and by providing nutrient supply and protection against infection and other hazards. Tear film complex composition contains proteins, such as enzymes, mucins, hormones, growth factors, neuropeptides, cytokines along with lipids, salts, and carbohydrates [2]. Ocular surface diseases carry profound variations on tear contents. Tears can be easily accessed and collected through minimally invasive methods; thus its analysis represents a promising approach for diagnosis and monitoring of human ocular surface diseases [3].

Proteomic analysis of human fluids has become one of the most relevant approaches for disease biomarkers research [4]. Proteome patterns in tears offer a powerful analytical tool to understand proper protein function in homeostasis as well as in underlying disease processes, and to provide biomarkers. Several studies have previously investigated tear proteomics in ocular diseases such as dry eye [5–7], keratoconus [8–10], and graft-versus-host-disease (GVHD) [11].

Three distinct ocular conditions were chosen for the tear proteomic comparison: keratoconus, severe dry eye related to graft-versus-host-disease (GVHD), and pterygium. Keratoconus is a primary corneal ectatic disease associated with progressive stromal thinning and protrusion leading to visual impairment. Prevalence varies from 8.8 to 229, and reported incidence ranges from 1.3 to 25 per 100.000 per year [12, 13]. Dry Eye Disease (DED) is a common, complex and multifactorial disease of the ocular surface and tear film that results in discomfort and visual disturbance [14]. Severe forms such as seen in chronic GVHD are a major complication after allogeneic stem cell transplantation and can lead to significant morbidity [15]. Pterygium is an ocular surface disorder with a higher incidence in tropical climates, consisting of a non-neoplastic elastotic degeneration of the bulbar conjunctiva that extends to the corneal surface, and is mainly associated to long-term ultraviolet radiation exposure [16].

All these conditions—keratoconus, pterygium, and chronic GVHD dry eye—can significantly alter the ocular surface and tear film parameters [15, 17, 18]. This pilot study aimed to compare the tear proteomic profile in these distinct ocular disorders and report possible biomarkers.

## Methods

The study was carried out with the approval of the Institutional Research Ethics Committee Board and was conducted under the tenets of the Declaration of Helsinki and current legislation on clinical research. Written informed consent was obtained from all subjects after explanation of the procedures and study requirements.

A total of 29 study subjects were recruited at the Ambulatory of Ophthalmology, Clinical Hospital of the University of Campinas (UNICAMP).

Study subjects were divided into four groups: 4 patients with keratoconus, 9 patients with pterygium, 10 patients with GVHD, and 6 normal controls. Each participant was submitted to a broad clinical examination, including ocular surface evaluation and corneal tomographic imaging. Keratoconus diagnosis was confirmed by imaging evaluation showing characteristic corneal steepening, thinning, altered corneal elevation maps, and irregular astigmatism [13]. Pterygium diagnosis was based on the clinical presentation at slip lamp examination of a fibrovascular proliferation of the bulbar conjunctiva related to irritative symptoms [19]. Dry-eye related chronic ocular GVHD was confirmed through a comprehensive evaluation of tear film and ocular surface parameters, such as tear film break up time, Schirmer test, corneal staining, tear meniscus height, in patients with prior hematopoietic stem cell transplantation [15]. Inclusion criteria for the control group were corneal tomographic maps and indices within the normal range, ocular surface parameters within the normal range, and no clinical sign of pterygium or any other ocular surface disease.

Tear samples were collected using a micropipette after a flush of sterile distilled water (20 µL) over the eye surface, and then they were transferred to Eppendorf tubes and frozen at − 80 °C.

### Sample preparation

Tear samples were thawed in ice, and a final volume of 15 µL was used for digestion. In sequence, we added a volume 1:1 of urea 8 M. Samples were reduced with the addition of 5 mM final concentration of DTT (DL-Dithiothreitol—Sigma-Aldrich®) and incubated for 25 min at 56 °C, and then alkylated with 14 mM final concentration of IAA (Iodoacetamide—SigmaAldrich®), for 30 min at room temperature and in the dark. After these steps, we added 1 mM of CaCl^2^ (Synth®), followed by digestion with 0.3 µg of trypsin (Sequencing Grade Modified Trypsin, V5111, Promega) for 16 h at 37 °C. After digestion with trypsin, its reaction was interrupted with the addition of formic acid at 1% (Merck®), with a pH of less than 3. In sequence, samples were desalted with Stage Tips with C18 membranes (Octadecyl C18-bonded silica—3 M Empore™ extraction disks) and then completely dried (SPD 1010 SpeedVac®, Thermo) [20].

### Liquid chromatography–mass spectrometry (LC–MS) sample injection

A 2 µL aliquot from each sample was analyzed in the mass spectrometer LTQ Orbitrap Velos (Thermo Fisher Scientific) coupled with the liquid chromatography system EASYnLC II (Proxeon) through a nanoelectrospray interface. Peptides were separated by a 2–90% acetonitrile gradient in 0.1% formic acid using an analytical PicoFrit Column (20 cm × ID75 μm, 5 μm particle size, New objective) at a flow rate of 300 nL/min over 80 min. The nanoelectrospray voltage was set to 2.2 kV, and the source temperature was 275 °C. The 20 most intense ions were chosen for CID collision-induced dissociation (CID) fragmentation, based on a data-dependent analysis. The full scan mass spectrometry (MS) spectra (m/z 300–1600) were acquired in the Orbitrap analyzer after accumulation to a target value of 1e6. The resolution in the Orbitrap was set to r = 60,000, and the most intense peaks were fragmented by CID with a normalized collision energy of 35% and activation time of 10 ms. The signal threshold for triggering an MS/MS event was set to 1000 counts, with a dynamic exclusion of 60 s.

### Pre-processing

After data acquisition, we performed data processing with the Andromeda algorithm within the MaxQuant version 1.3.0.3 software against the UniProt Human Protein Database (Release: March 2017, 92,934 sequences and 36,874,315 residues).

Bioinformatic analysis was performed using Perseus version 1.5.1.6 software. We used logarithmic transformation and application of filters to exclude proteins with reverse sequences, proteins identified by only one modified peptide, and filtering by minimum valid values of 5 in at least one group.

### Statistical analysis

MS data were log2 transformed before statistical analysis. Univariate analyses were performed on GraphPad Prism version 6.00. Samples measurements from patients with GVHD, pterygium or keratoconus were compared to respective control samples by Student's t-test, not paired and without or with correction for multiple analyses (FDR 5%, FDR 1% or Holm-Sidak method). Multivariate analyses were performed on online platform Metaboanalyst (https://www.metaboanalyst.ca). Top 10 features for pterygium and top 8 features from VIP-PLSDA (Variable Importance in Projection in Partial Least Scores Discriminant Analysis) score for GVHD and keratoconus were selected for heat map visualization, clustering and receiver operating characteristic (ROC) analyses. For heat map visualization, data was auto-scaled. Distance measurement was Euclidean, and clustering algorithm was Ward. The area under curve (AUC) from multivariate ROC analyses and corresponding to 95% confidence intervals were calculated to estimate the clinical potential of selected metabolites as biomarkers [21].

### Biochemical pathways prospection

Proteins with p-value < 0.05 in Student’s t-test analyses were selected, and biochemical pathways prospections were performed for each disease on KEGG Mapper/Search Pathway (https://www.kegg.jp/kegg/tool/map_pathway1.html), last updated: June 10, 2014) against *Homo sapiens* database [22].

## Results

Clinical characteristics of each study group are presented in Table 1.

**Table 1:** Clinical characteristics of each study group

Group	n	Age (years, mean)	Sex (female:male ratio)
Keratoconus	4	30.5	2:2
Pterygium	9	47.2	3:6
GVHD	10	49.6	7:3
Control	6	47.5	5:1

### Proteins identification and quantification

**Fig. 1:**
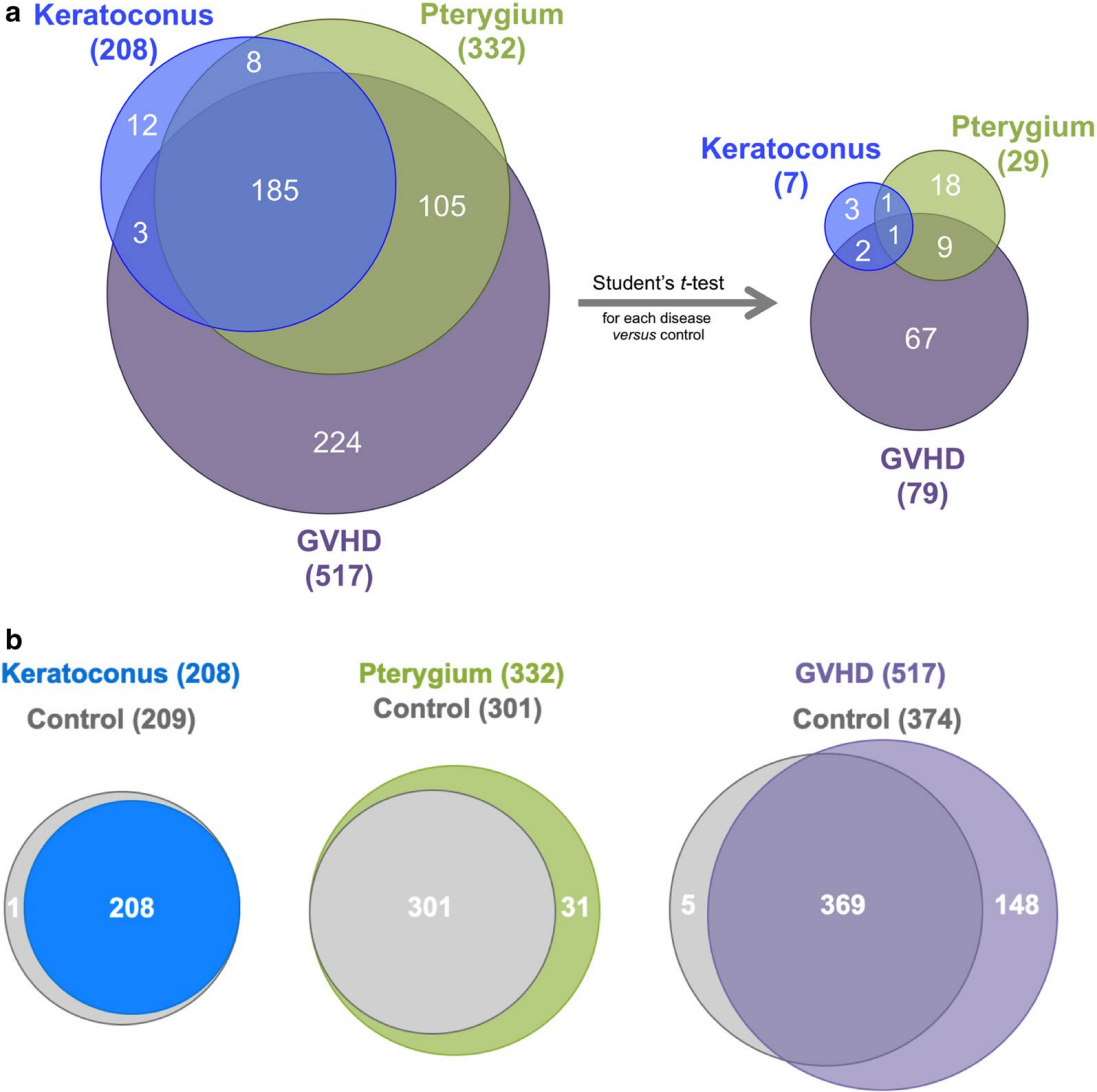
**a**, **b** Relationship between keratoconus, pterygium, and GVHD tear proteomes. **a** Number of proteins identified for each disease to the left, and number of differentially expressed proteins within statistical significance (p < 0.05) to the right. **b** Relationship between total number of identified proteins for each disease and control group

The MS quantification analysis identified a total of 208 distinct proteins in the tear samples from keratoconus group, 332 proteins in the pterygium group, and 517 proteins in the GVHD group (Additional file 1: Table S1; Additional file 2: Table S2; Additional file 3: Table S3). The relationship between the tear proteomes analyzed in our study is shown in Fig. 1a, b. The total number of distinct proteins identified for each disease is shown, and Venn diagram displays the number of overlapping proteins in the three proteomes, in which proteins in common are shown in the intersection between the circles. As can be observed in Fig. 1b, the total number of identified proteins for the control group differ between groups, because in the spectra preprocessing stage each disease was analyzed separately. This is necessary to avoid interference of a specific disease in another and artifact production. In this process, FDR is applied for each comparison causing slight differences in the number of identified proteins. Consequently, different numbers of proteins were found for each pair of disease versus control group. This also prevents the inclusion of the control group in the three disease’s Venn diagram in Fig. 1a.

### Biochemical pathways prospection

After t-test statistical analyses, 7 proteins were found with increased levels in the keratoconus group comparing to controls, as shown in Table 2. None of these proteins retained statistical significance after multiple comparisons correction. The analysis did not show any protein with decreased levels in the keratoconus group.

**Table 2:** Student’s t-test analysis of tear proteome from patients diagnosed with keratoconus *versus* control

Gene name	Protein	p-value	FDR 5%	FDR 1%	FWER	Control average LFQ log transformed	Keratoconus average LFQ log transformed	SE of difference
IGHV5-10–1; IGHV5-51	Immunoglobulin heavy variable 5–10-1; Immunoglobulin heavy variable 5–51	3.00E−03	N	N	N	21.8	23.2	0.3
PRR27	Proline-rich protein 27	3.10E−03	N	N	N	22.3	25.4	0.7
IGHM	Ig mu chain C region; Ig mu heavy chain disease protein	4.49E−03	N	N	N	26.0	27.8	0.4
HIST1H2BA§	Histone H2B type 1-A	4.68E−03	N	N	N	21.1	22.9	0.4
KRT13	Keratin, type I cytoskeletal 13	2.06E−02	N	N	N	25.5	27.4	0.7
IGHV3-23#	Immunoglobulin heavy variable 3–23	3.58E−02	N	N	N	24.5	25.5	0.4
DEFA1; DEFA3	Neutrophil defensin 1; HP 1–56; Neutrophil defensin 2; Neutrophil defensin 3; HP 3–56; Neutrophil defensin 2	3.69E−02	N	N	N	25.2	27.9	1.1

Proteins with increased abundance in keratoconus in comparison with controls are shown

FDR: false discovery rate. FEWR: family-wise error rate (Holm-Sidak method). Y: yes, still significant after multiple comparisons correction. N: no, not significant after multiple comparisons correction. SE: standard error. § histone cluster (HIST1H2BA; HIST1H2BK; HIST1H2BJ; HIST1H2BO; HIST1H2BB; H2BFS; HIST1H2BD; HIST1H2BC; HIST2H2BE; HIST2H2BF; HIST3H2BB; HIST1H2BH; HIST1H2BN; HIST1H2BM; HIST1H2BL). # immunoglobulin cluster (IGHV3-23; IGHV3-30; IGHV3-30–5)

After Student’s t-test analyses comparing pterygium group versus control, 29 proteins showed altered expression, 9 with decreased levels and 20 with increased levels comparing to controls, as shown in Tables 3 and 4. After multiple comparisons correction, 2 proteins with increased levels retained statistical significance with 5% false discovery rate (FDR) and 1 protein with family-wise error rate (FWER) using the Holm-Sidak method.

**Table 3:** Student’s t-test analysis of tear proteome from patients diagnosed with pterygium *versus* control

Gene name	Protein	p-value	FDR 5%	FDR 1%	FWER	Control average LFQ log transformed	Pterygium average LFQ log transformed	SE of difference
SERPINA1	Alpha-1-antitrypsin; Short peptide from AAT	2.55E−03	N	N	N	25.8	23.2	0.7
ORM1	Alpha-1-acid glycoprotein 1	3.01E−03	N	N	N	26.0	23.2	0.7
APOA1	Apolipoprotein A-I; Truncated apolipoprotein A-I	5.11E−03	N	N	N	27.3	23.3	1.1
SERPINA3	Alpha-1-antichymotrypsin	1.69E−02	N	N	N	25.2	23.6	0.6
IGHG2	Ig gamma-2 chain C region	3.17E−02	N	N	N	26.5	24.4	0.8
CTSD	Cathepsin D	4.27E−02	N	N	N	24.4	22.8	0.7
SERPINC1	Antithrombin-III	4.52E−02	N	N	N	25.9	24.0	0.8
PIP	Prolactin-inducible protein	4.90E−02	N	N	N	31.6	29.0	1.2
A1BG	Alpha-1B-glycoprotein	4.92E−02	N	N	N	23.4	21.9	0.6

Proteins with decreased abundance in pterygium in comparison with controls are shown

FDR: false discovery rate. FEWR: family-wise error rate (Holm-Sidak method). Y: yes, still significant after multiple comparisons correction. N: no, not significant after multiple comparisons correction. SE: standard error

**Table 4:** Student’s t-test analysis of tear proteome from patients diagnosed with pterygium *versus* control

Gene name	Protein	p-value	FDR 5%	FDR 1%	FWER	Control average LFQ log transformed	Pterygium average LFQ log transformed	SE of difference
KRT13	Keratin, type I cytoskeletal 13	1.16E−04	Y	N	Y	25.1	29.1	0.7
KRT14	Keratin, type I cytoskeletal 14	3.27E−04	Y	N	N	25.1	28.4	0.7
RPL11	60S ribosomal protein L11	1.76E−03	N	N	N	20.0	22.3	0.3
KRT5	Keratin, type II cytoskeletal 5	3.60E−03	N	N	N	26.7	29.3	0.7
UPK3BL; POLR2J3	Uroplakin-3b-like protein	6.69E−03	N	N	N	22.9	24.6	0.5
KRT6B	Keratin, type II cytoskeletal 6B	8.21E−03	N	N	N	25.7	27.9	0.7
S100A8	Protein S100-A8	8.43E−03	N	N	N	22.4	25.0	0.8
YWHAQ	14–3-3 protein theta	8.43E−03	N	N	N	21.1	22.9	0.4
ST13; ST13P4	Hsc70-interacting protein; Putative protein FAM10A4	1.12E−02	N	N	N	22.1	22.6	0.1
FLG2	Filaggrin-2	1.18E−02	N	N	N	20.7	24.6	1.2
S100A6	Protein S100-A6	1.53E−02	N	N	N	23.5	26.1	0.9
KRT4	Keratin, type II cytoskeletal 4	1.93E−02	N	N	N	25.4	27.6	0.8
HSPA8	Heat shock cognate 71 kDa protein	2.45E−02	N	N	N	22.0	23.7	0.6
MYL6; MYL6B	Myosin light polypeptide 6; Myosin light chain 6B	2.49E−02	N	N	N	22.1	23.6	0.6
ANXA2; ANXA2P2	Annexin A2; Annexin; Putative annexin A2-like protein	2.79E−02	N	N	N	25.1	26.6	0.6
UBC§	Polyubiquitin-C	3.26E−02	N	N	N	22.2	24.7	1.1
IGHV5-10–1	Immunoglobulin heavy variable 5–10-1	3.41E−02	N	N	N	21.9	23.1	0.5
CDC42	Cell division control protein 42 homolog	3.88E−02	N	N	N	21.1	22.1	0.4
YWHAZ	14–3-3 protein zeta/delta	4.12E−02	N	N	N	24.7	26.4	0.7
P4HB	Protein disulfide-isomerase	4.87E−02	N	N	N	21.9	23.0	0.5

Proteins with increased abundance in pterygium in comparison with controls are shown

FDR: false discovery rate. FEWR: family-wise error rate (Holm-Sidak method). Y: yes, still significant after multiple comparisons correction. N: no, not significant after multiple comparisons correction. § Ubiquitin cluster (UBC; UBB; RPS27A; UBA52). SE: standard error

After Student’s t-test analyses, 79 proteins showed altered expression in the GVHD group comparing to controls, 35 proteins with decreased levels and 44 with increased levels. Among the proteins with decreased levels, after multiple comparisons correction, 19 proteins retained statistical significance with 5% FDR, 6 proteins with 1% FDR, and 2 proteins with FWER using the Holm-Sidak method (Table 5). Among the proteins with increased levels, after multiple comparisons correction, 17 proteins retained statistical significance with 5% FDR, and 3 proteins with both 1% FDR and FWER using the Holm-Sidak method (Table 6).

**Table 5:** Student’s t-test analysis of tear proteome from patients diagnosed with ocular GVHD *versus* control

Gene name	Protein	p-value	FDR 5%	FDR 1%	FWER	Control average LFQ log transformed	GVHD average LFQ log transformed	SE of difference
LCN1	Lipocalin-1	6.92E−05	Y	Y	Y	34.6	25.2	1.7
TGM2	Protein-glutamine gamma-glutamyltransferase 2	9.48E−05	Y	Y	Y	24.2	21.7	0.4
LTF	Lactotransferrin; Kaliocin-1; Lactoferroxin-A; Lactoferroxin-B; Lactoferroxin-C	1.97E−04	Y	Y	N	35.4	28.6	1.4
AZGP1	Zinc-alpha-2-glycoprotein	2.29E−04	Y	Y	N	32.3	28.9	0.7
CRISP3	Cysteine-rich secretory protein 3	2.39E−04	Y	Y	N	27.4	21.8	0.6
IGJ	Immunoglobulin J chain	2.83E−04	Y	Y	N	29.8	25.9	0.8
CTSB	Cathepsin B; Cathepsin B light chain; Cathepsin B heavy chain	3.80E−04	Y	N	N	25.4	22.7	0.6
PIGR	Polymeric immunoglobulin receptor; Secretory component	4.62E−04	Y	N	N	30.8	27.3	0.8
LYZ	Lysozyme C	6.12E−04	Y	N	N	33.4	28.2	1.2
LGALS3BP	Galectin-3-binding protein	6.57E−04	Y	N	N	25.0	22.1	0.6
NUCB2	Nucleobindin-2	7.02E−04	Y	N	N	24.7	21.8	0.5
DMBT1	Deleted in malignant brain tumors 1 protein	8.31E−04	Y	N	N	29.4	24.7	1.1
PIP	Prolactin-inducible protein	1.87E−03	Y	N	N	31.3	26.6	1.2
IGHA2	Immunoglobulin heavy constant alpha 2	1.99E−03	Y	N	N	27.4	24.6	0.7
FN1	Fibronectin; Anastellin; Ugl-Y1; Ugl-Y2; Ugl-Y3	2.07E−03	Y	N	N	27.7	24.2	0.9
MSLN	Mesothelin; Megakaryocyte-potentiating factor; Mesothelin, cleaved form	2.44E−03	Y	N	N	25.7	22.2	0.9
CLU	Clusterin; Clusterin beta chain; Clusterin alpha chain; Clusterin	4.16E−03	Y	N	N	27.3	25.4	0.6
IGHA1	Ig alpha-1 chain C region	5.33E−03	Y	N	N	32.5	30.0	0.7
B2M	Beta-2-microglobulin; Beta-2-microglobulin form pI 5.3	5.59E−03	Y	N	N	28.2	24.9	1.0
DBNL	Drebrin-like protein	6.22E−03	N	N	N	21.5	20.5	0.3
PRR4	Proline-rich protein 4	7.24E−03	N	N	N	31.1	24.5	2.0
IGHA2	Ig alpha-2 chain C region	7.43E−03	N	N	N	26.2	23.2	0.5
PSAP	Proactivator polypeptide; Saposin-A; Saposin-B-Val; Saposin-B; Saposin-C; Saposin-D	7.65E−03	N	N	N	24.0	22.7	0.4
SLPI	Antileukoproteinase	9.52E−03	N	N	N	27.1	24.8	0.8
CBR1	Carbonyl reductase [NADPH] 1	1.50E−02	N	N	N	22.7	20.8	0.6
TUBB4B; TUBB4A	Tubulin beta-4B chain; Tubulin beta-4A chain	1.65E−02	N	N	N	27.0	25.2	0.6
RNH1	Ribonuclease inhibitor	1.69E−02	N	N	N	23.0	21.9	0.3
HNRNPK	Heterogeneous nuclear ribonucleoprotein K	2.08E−02	N	N	N	24.1	20.6	1.1
PRDX6	Peroxiredoxin-6	2.94E−02	N	N	N	23.9	22.0	0.7
IGHV3-7@	Immunoglobulin heavy variable 3–7	3.30E−02	N	N	N	22.6	21.0	0.7
CST3	Cystatin-C	3.94E−02	N	N	N	24.0	21.6	1.0
PHGDH	D-3-phosphoglycerate dehydrogenase	4.14E−02	N	N	N	21.0	19.0	0.6
IGHV3-72	Immunoglobulin heavy variable 3–72	4.17E−02	N	N	N	23.4	22.4	0.5
IGHV3OR16-12	Immunoglobulin heavy variable 3/OR16-12 (non-functional)	4.34E−02	N	N	N	23.5	21.8	0.7
IGKV2D-24; IGKV2-24	Immunoglobulin kappa variable 2D-24 (non-functional), Immunoglobulin kappa variable 2–24	4.71E−02	N	N	N	22.9	22.1	0.3

Proteins with decreased abundance in GVHD in comparison with controls are shown

FDR: false discovery rate. FEWR: family-wise error rate (Holm-Sidak method). Y: yes, still significant after multiple comparisons correction. N: no, not significant after multiple comparisons correction. SE: standard error. @ Immunoglobulin cluster (P01780; A0A0B4J1V1; P01762; P01763; A0A0C4DH32; A0A075B7F0)

**Table 6:** Student’s t-test analysis of tear proteome from patients diagnosed with ocular GVHD versus control

Gene name	Protein	p-value	FDR 5%	FDR 1%	FWER	Control average LFQ log transformed	GVHD average LFQ log transformed	SE of difference
TKT	Transketolase	2.75E−05	Y	Y	Y	21.2	25.4	0.6
DEFA1; DEFA3	Neutrophil defensin 1; HP 1–56; Neutrophil defensin 2; Neutrophil defensin 3; HP 3–56; Neutrophil defensin 2	3.90E−05	Y	Y	Y	24.7	30.1	0.9
KRT13§	Keratin, type I cytoskeletal 13	5.89E−05	Y	Y	Y	25.8	30.1	0.8
KRT4	Keratin, type II cytoskeletal 4	6.68E−04	Y	N	N	25.5	28.9	0.8
KRT5	Keratin, type II cytoskeletal 5	7.04E−04	Y	N	N	26.2	29.2	0.7
SAA4	Serum amyloid A-4 protein	1.49E−03	Y	N	N	22.6	23.9	0.3
CASP14	Caspase-14; Caspase-14 subunit p19; Caspase-14 subunit p10	2.43E−03	Y	N	N	22.2	25.2	0.7
ANXA1	Annexin A1; Annexin	2.47E−03	Y	N	N	24.8	27.1	0.6
KRT19	Keratin, type I cytoskeletal 19	2.48E−03	Y	N	N	25.6	28.5	0.8
A2M	Alpha-2-macroglobulin	2.89E−03	Y	N	N	26.1	29.0	0.8
CFB	Complement factor B; Complement factor B Ba fragment; Complement factor B Bb fragment	3.20E−03	Y	N	N	24.2	26.9	0.7
KRT6A; KRT6C	Keratin, type II cytoskeletal 6A; Keratin, type II cytoskeletal 6C	3.74E−03	Y	N	N	28.1	30.6	0.7
SPRR3	Small proline-rich protein 3	4.04E−03	Y	N	N	22.3	24.9	0.7
PLG	Plasminogen	4.67E−03	Y	N	N	22.7	24.7	0.5
IGHM	Ig mu chain C region; Ig mu heavy chain disease protein	4.86E−03	Y	N	N	26.3	28.2	0.6
ANXA2; ANXA2P2	Annexin A2; Annexin; Putative annexin A2-like protein	5.22E−03	Y	N	N	25.1	27.7	0.8
HRG	Histidine-rich glycoprotein	5.85E−03	Y	N	N	21.7	23.8	0.6
H2AFX#	Histone H2AX	6.76E−03	N	N	N	22.0	24.4	0.7
FLG	Filaggrin	6.93E−03	N	N	N	21.8	25.7	1.2
MYL6	Myosin light polypeptide 6	8.11E−03	N	N	N	21.9	24.0	0.7
IGHG3	Ig gamma-3 chain C region	9.38E−03	N	N	N	27.4	29.8	0.8
CTSG	Cathepsin G	9.40E−03	N	N	N	20.3	24.6	1.3
HIST1H4A	Histone H4	1.00E−02	N	N	N	23.2	25.2	0.6
CFH	Complement factor H	1.22E−02	N	N	N	24.2	26.4	0.7
YWHAZ	14–3-3 protein zeta/delta	1.25E−02	N	N	N	24.7	26.6	0.6
EZR	Ezrin	1.80E−02	N	N	N	24.6	25.9	0.5
HSPA8	Heat shock cognate 71 kDa protein	1.94E−02	N	N	N	23.2	25.0	0.7
ACTN4	Alpha-actinin-4	1.98E−02	N	N	N	22.6	25.0	0.9
LRG1	Leucine-rich alpha-2-glycoprotein	2.05E−02	N	N	N	21.9	23.2	0.5
S100A14	Protein S100-A14	2.09E−02	N	N	N	19.5	21.7	0.8
KRT14	Keratin, type I cytoskeletal 14	2.30E−02	N	N	N	24.8	27.8	1.1
C4BPA	C4b-binding protein alpha chain	2.41E−02	N	N	N	21.6	23.7	0.8
TF	Serotransferrin	2.41E−02	N	N	N	28.3	30.9	1.0
YWHAB	14–3-3 protein beta/alpha; 14–3-3 protein beta/alpha, N-terminally processed	2.51E−02	N	N	N	23.7	25.3	0.6
ITIH1	Inter-alpha-trypsin inhibitor heavy chain H1	2.58E−02	N	N	N	21.8	22.9	0.4
C3Φ	Complement C3	2.61E−02	N	N	N	27.6	29.2	0.6
TFF1	Trefoil factor 1	2.65E−02	N	N	N	22.9	25.8	1.1
ALB	Serum albumin	2.82E−02	N	N	N	33.5	35.3	0.7
ACTB	Actin, cytoplasmic 1; Actin, cytoplasmic 1, N-terminally processed	3.14E−02	N	N	N	27.3	29.0	0.7
CFL1	Cofilin-1	3.62E−02	N	N	N	21.9	24.0	0.9
KNG1	Kininogen-1	4.37E−02	N	N	N	23.8	25.3	0.7
S100A9	Protein S100-A9	4.40E−02	N	N	N	24.6	26.6	0.9
UPK3BL; POLR2J3	Uroplakin-3b-like protein	4.44E−02	N	N	N	21.9	23.0	0.5
TXN	Thioredoxin	4.96E−02	N	N	N	22.9	24.7	0.8

Proteins with increased abundance in GVHD in comparison with controls are shown

FDR: false discovery rate. FEWR: family-wise error rate (Holm-Sidak method). Y: yes, still significant after multiple comparisons correction. N: no, not significant after multiple comparisons correction. SE: standard error. § keratin cluster (P13646-1; K7EQH6; P35900; P35900; K7EMJ2; A0A140TA69; Q8IUT8; O76011; Q6IFU5; Q6A162; O76009; Q6NTB9; O76009; A0A140TA62; O76011; G3V1C2; J3QR55). # histone cluster (H2AFX; HIST1H2AA; HIST1H2AB; HIST1H2AG; HIST1H2AD; HIST2H2AA3; HIST3H2A; HIST1H2AC; HIST2H2AC; H2AFJ; HIST1H2AH; HIST1H2AJ; HIST2H2AB; H2AFZ; H2AFV). Φ complement cluster (P01024; Q2UVX4; M0QXZ3; M0QYC8; M0R0Q9)

Figure 1a (to the right) shows the number of differentially expressed proteins with p < 0.05 after Student’s t-test analyses in the three disease groups compared to controls. There is one protein in common between all groups (Keratin, type I cytoskeletal 13, increased level); another protein in common between keratoconus and pterygium group (Immunoglobulin heavy variable 5-10-1, increased level); 2 more proteins in common between keratoconus and GVHD group (Neutrophil defensin and Immunoglobulin mu chain C region, increased levels); and another 9 proteins in common between GVHD and pterygium groups (Keratin, type I cytoskeletal 14, Keratin, type II cytoskeletal 5, Keratin, type II cytoskeletal 4, Uroplakin-3b-like protein, Heat shock cognate 71 kDa protein, Myosin light polypeptide 6, Annexin A2, 14-3-3 protein zeta/delta, increased levels, and Prolactin-inducible protein, decreased level).

**Fig. 2:**
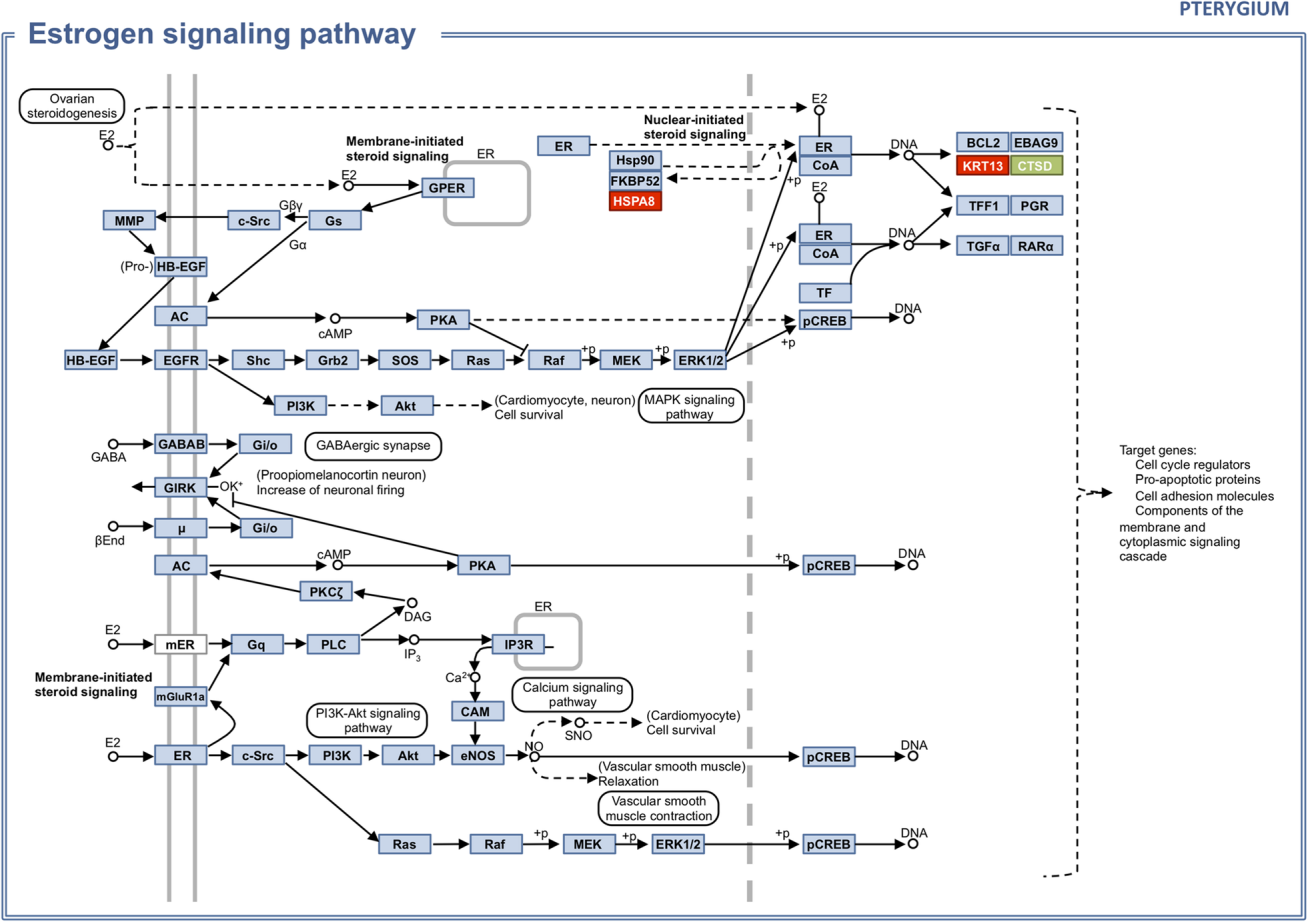
Estrogen signaling pathway with altered proteins in pterygium tear proteome in highlight. Red: increased quantification. Green: decreased quantification

The biochemical pathway prospection performed on the KEGG mapper for the pterygium tear proteome is shown in Fig. 2, which represents the estrogen signaling pathway, with altered proteins highlighted: increased levels of KRT13 (Keratin, type I cytoskeletal 13) and HSPA8 (Heat shock cognate 71 kDa protein), and decreased level of CTSD (Cathepsin D).

**Fig. 3:**
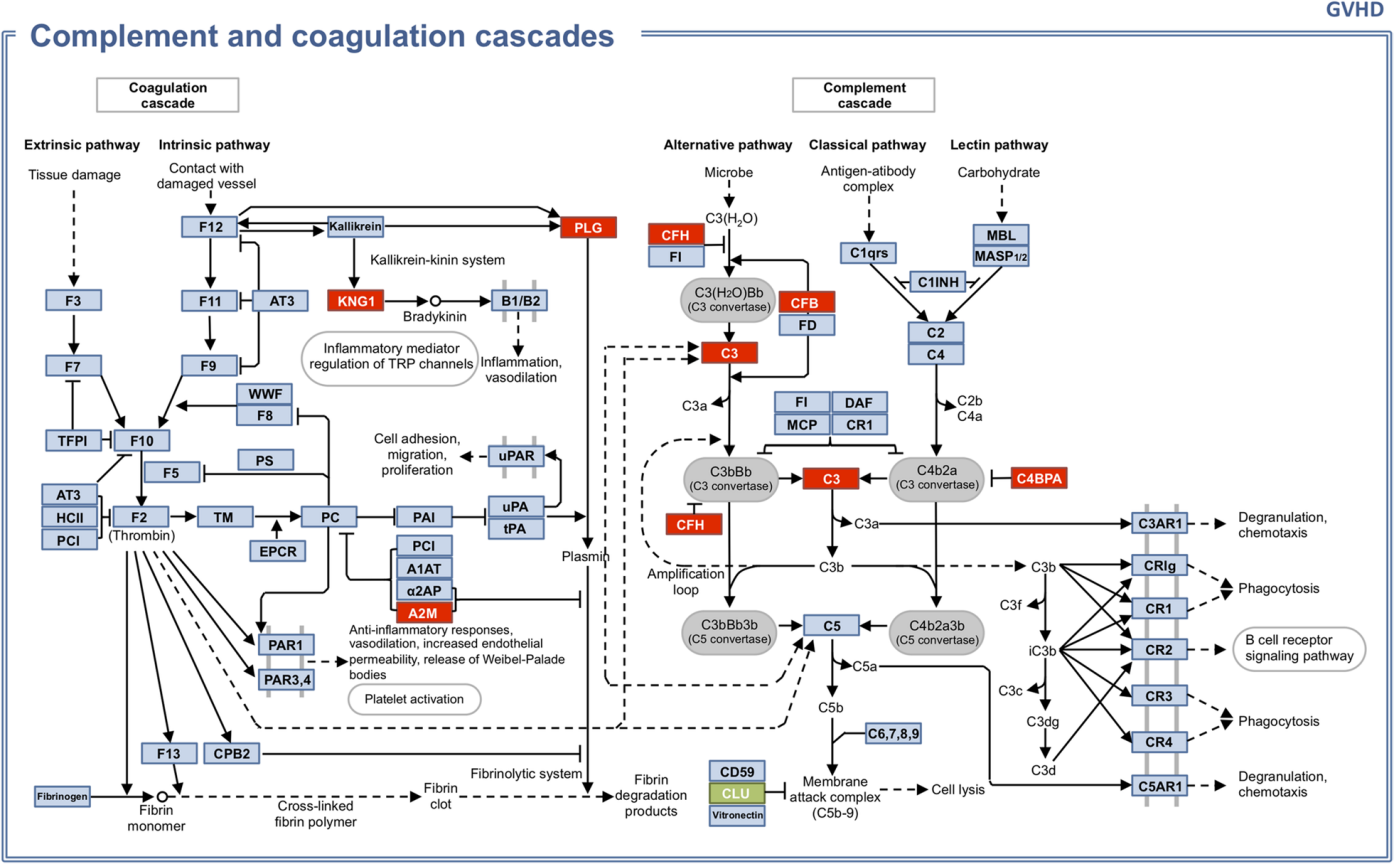
Complement and coagulation cascades with altered proteins in GVHD tear proteome in highlight. Red: increased quantification. Green: decreased quantification

From GVHD tear protein profile, biochemical pathway prospection retrieved the complement and coagulation cascades (Fig. 3), with altered proteins highlighted: increased levels of KNG1 (Kininogen-1), A2M (Alpha-2-macroglobulin), PLG (Plasminogen), CFH (Complement factor H), CFB (Complement factor B), C3 (Complement C3), and C4BPA (C4b-binding protein alpha chain), and decreased level of CLU (Clusterin).

Biochemical pathway prospection for keratoconus did not yield significant results because of the low number of statistically significant proteins between keratoconus and control group in the univariate analysis.

### Potential biomarkers

**Fig. 4:**
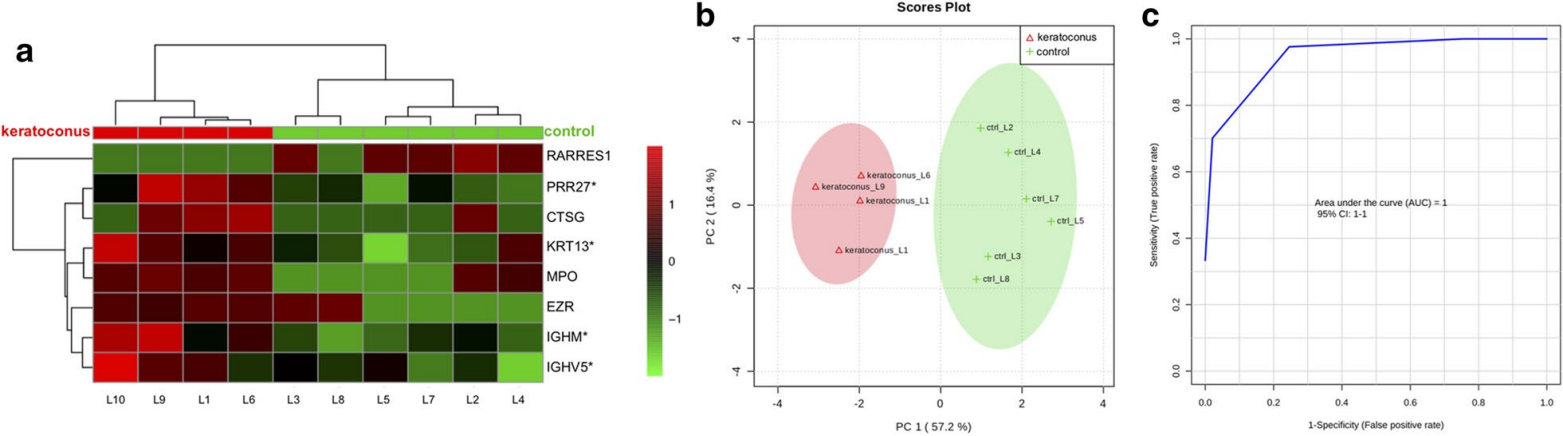
Heat map, PCA scores plot, and ROC curve for keratoconus group. **a** Heat map, the colors indicate relative expression levels, with red colors for increased protein levels, and green colors for decreased protein levels. **b** PCA (principal component analysis) scores plot. **c** ROC (receiver operating characteristic) curve

**Fig. 5:**
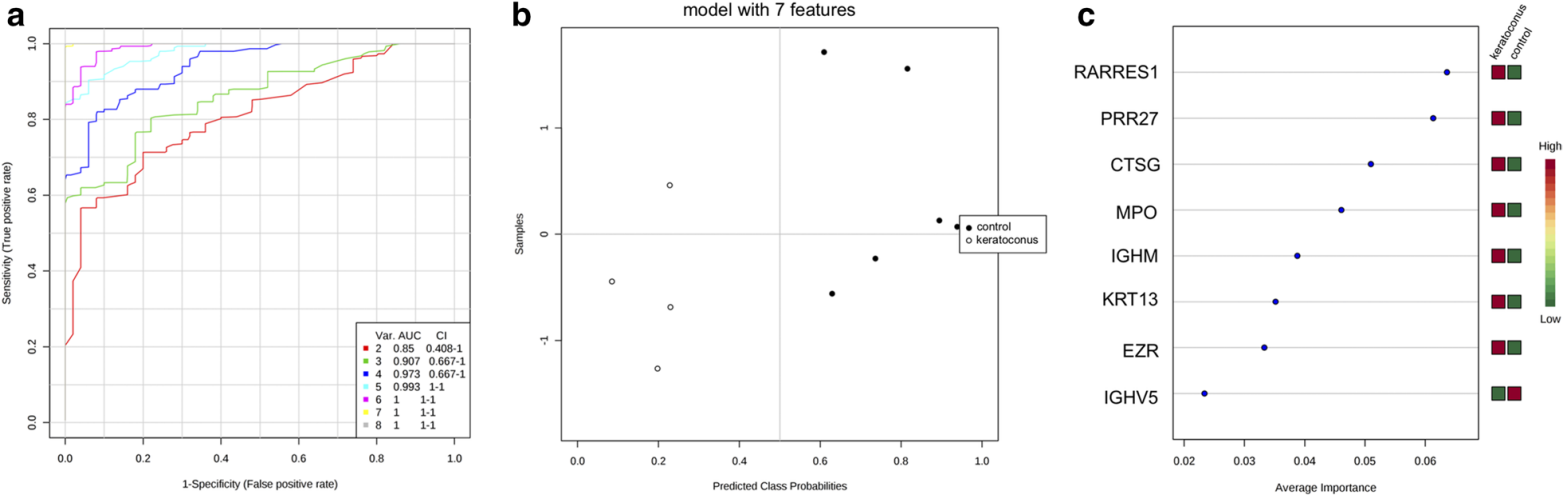
Multivariate ROC analysis and potential biomarkers average importance for keratoconus group. **a** Area under curve (AUC) from multivariate ROC analyses and corresponding 95% confidence intervals (CI). **b** Predicted Class Probabilities. **c** Potential biomarkers displayed in order of average importance

Heat map dendrographic profiles, PCA (principal component analysis) scores plot, and ROC (receiver operating characteristic) curves for the keratoconus group are shown in Fig. 4. PCA scores were 57.2% for PC1 (principal component) and 16.4% for PC2. The area under curve (AUC) from multivariate ROC analyses and corresponding 95% confidence intervals are shown in Fig. 5. After these multivariate analyses, the top 8 features from VIP-PLSDA were chosen, the area under curve (AUC) from multivariate ROC analyses and corresponding to 95% confidence intervals were calculated, and the proteins identified as potential biomarkers are presented in decreasing order of average importance on Table 7.

**Table 7:** Potential biomarkers in tears for keratoconus

Protein	Full name
RARRES1	Retinoic acid receptor responder protein 1
PRR27	Proline-rich protein 27
CTSG	Cathepsin G
MPO	Myeloperoxidase
IGHM	Immunoglobulin heavy constant mu
KRT13	Keratin, type I cytoskeletal 13
EZR	Ezrin
IGHV5	Immunoglobulin heavy variable 5

Biomarkers chosen after VIP-PLSDA (Variable Importance in Projection in Partial Least Scores Discriminant Analysis) multivariate analysis. Decreasing order of average importance

**Fig. 6:**
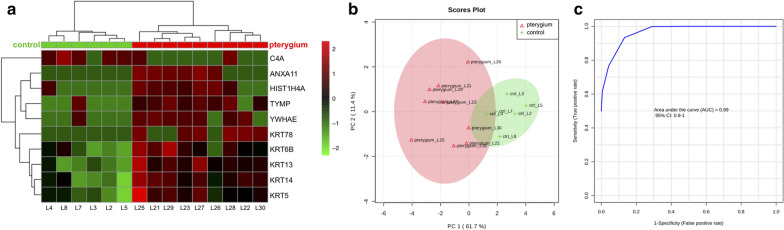
Heat map, PCA scores plot, and ROC curve for pterygium group. **a** Heat map, the colors indicate relative expression levels, with red colors for increased protein levels, and green colors for decreased protein levels. **b** PCA (principal component analysis) scores plot. **c** ROC (receiver operating characteristic) curve

**Fig. 7:**
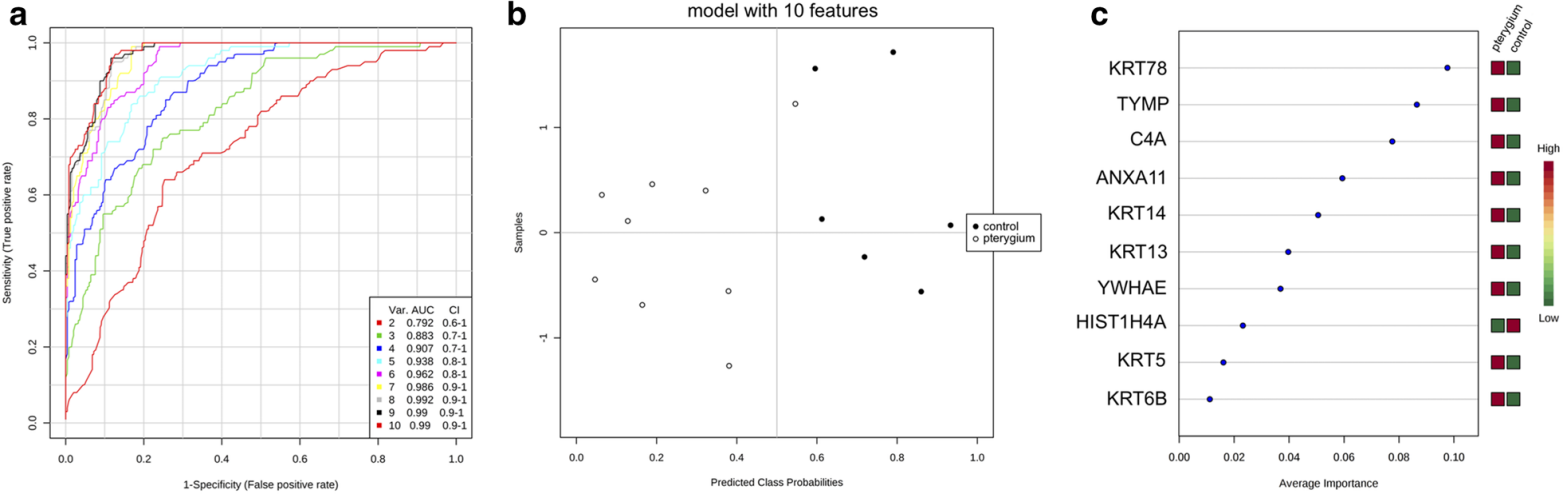
Multivariate ROC analysis and potential biomarkers average importance for pterygium group. **a** Area under curve (AUC) from multivariate ROC analyses and corresponding 95% confidence intervals (CI). **b** Predicted Class Probabilities. **c** Potential biomarkers displayed in order of average importance

Heat map dendrographic profiles, PCA scores plot, and ROC curves for pterygium group are shown in Fig. 6. PCA scores were 61.7% for PC1 and 11.4% for PC2. The area under curve from multivariate ROC analyses and corresponding 95% confidence intervals are shown in Fig. 7. After these multivariate analyses, the top 10 features from VIP-PLSDA were chosen, the area under curve (AUC) from multivariate ROC analyses and corresponding to 95% confidence intervals were calculated, and the proteins identified as potential biomarkers are presented in decreasing order of average importance on Table 8.

**Table 8:** Potential biomarkers in tears for pterygium

Protein	Full name
KRT78	Keratin, type II cytoskeletal 78
TYMP	Thymidine phosphorylase
C4A	Complement C4-A
ANXA11	Annexin A11
KRT14	Keratin, type I cytoskeletal 14
KRT13	Keratin, type I cytoskeletal 13
YWHAE	14–3-3 protein epsilon
HIST1H4A	Histone H4
KRT5	Keratin, type II cytoskeletal 5
KRT6B	Keratin, type II cytoskeletal 6B

Biomarkers chosen after VIP-PLSDA (Variable Importance in Projection in Partial Least Scores Discriminant Analysis) multivariate analysis. Decreasing order of average importance

**Fig. 8:**
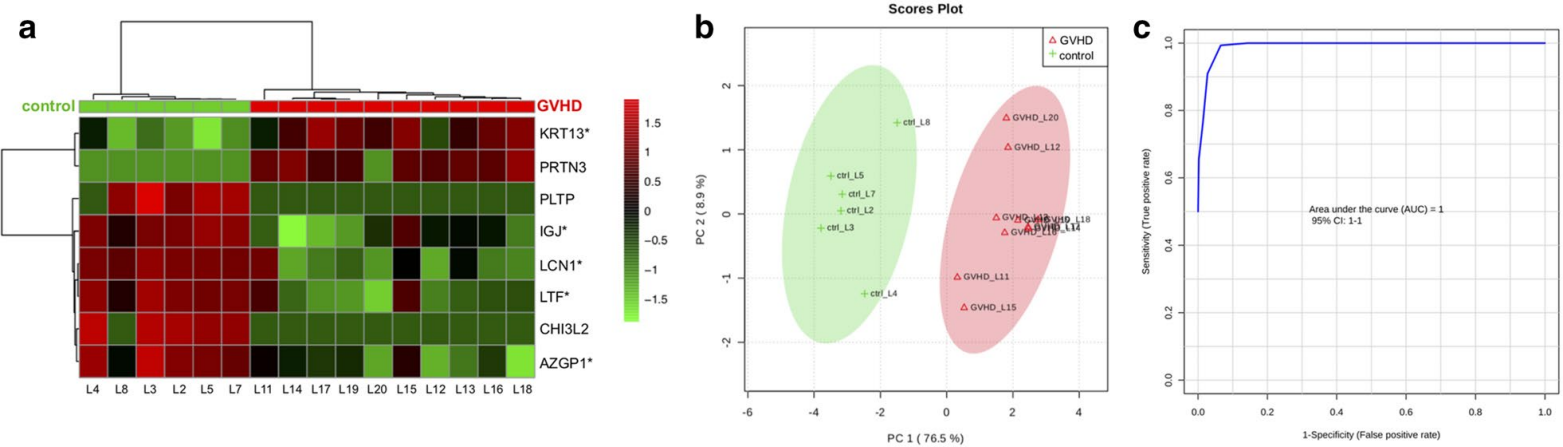
Heat map, PCA scores plot, and ROC curve for GVHD group. **a** Heat map, the colors indicate relative expression levels, with red colors for increased protein levels, and green colors for decreased protein levels. **b** PCA (principal component analysis) scores plot. **c** ROC (receiver operating characteristic) curve

**Fig. 9:**
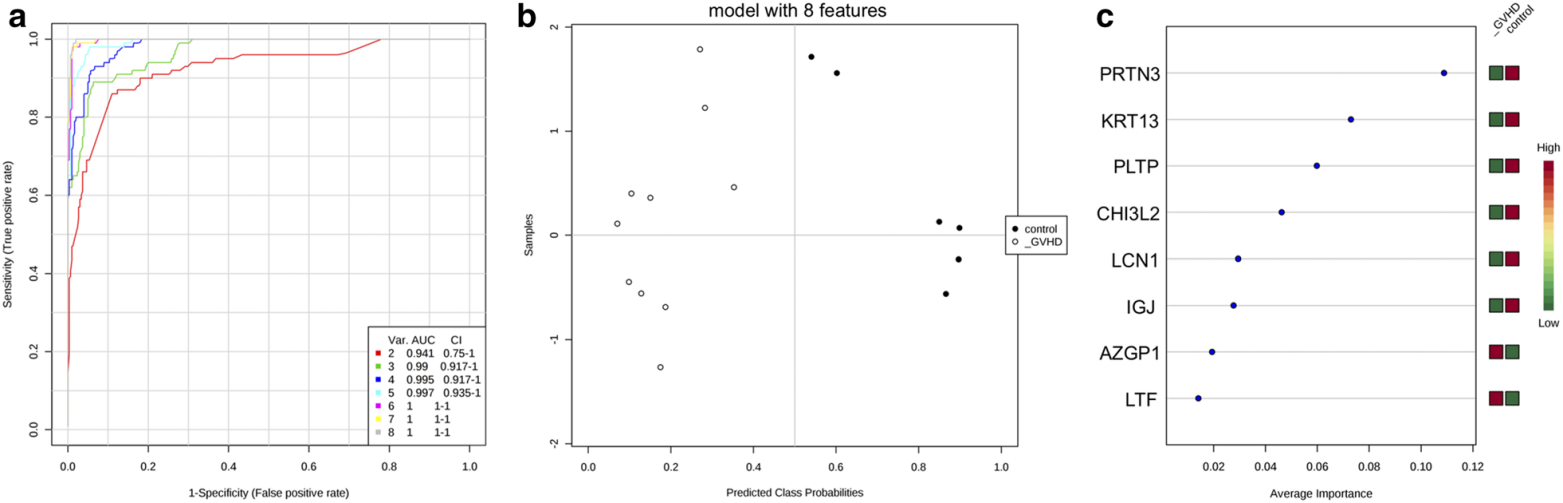
Multivariate ROC analysis and potential biomarkers average importance for GVHD group. **a** Area under curve (AUC) from multivariate ROC analyses and corresponding 95% confidence intervals (CI). **b** Predicted Class Probabilities. **c** Potential biomarkers displayed in order of average importance

Heat map dendrographic profiles, PCA scores plot, and ROC curves for the GVHD group are shown in Fig. 8. PCA scores were 76.5% for PC1 and 8.9% for PC2. The area under curve from multivariate ROC analyses and corresponding 95% confidence intervals are shown in Fig. 9. After these multivariate analyses, the top 8 features from VIP-PLSDA were chosen, the area under curve (AUC) from multivariate ROC analyses and corresponding to 95% confidence intervals were calculated, and the proteins identified as potential biomarkers are presented in decreasing order of average importance on Table 9.

**Table 9:** Potential biomarkers in tears for GVHD related dry eye

Protein	Full name
PRTN3	Myeloblastin
KRT13	Keratin, type I cytoskeletal 13
PLTP	Phospholipid transfer protein
CHI3L2	Chitinase-3-like protein 2
LCN1	Lipocalin-1
JCHAIN	Immunoglobulin J chain
AZGP1	Zinc-alpha-2-glycoprotein
LTF	Lactotransferrin

Biomarkers chosen after VIP-PLSDA (Variable Importance in Projection in Partial Least Scores Discriminant Analysis) multivariate analysis. Decreasing order of average importance

## Discussion

Tear film of three different ocular diseases—keratoconus, pterygium, and chronic GVHD related dry eye – were analyzed using LC–MS for quantitative proteomic investigation. Each group was compared to a control group, and each disease displayed distinct proteome profile.

Although classically described as a non-inflammatory disease [13, 23], recent research has shown altered inflammatory pathways and mediators in keratoconus corneas and tear film [9, 10, 24–27]. Despite extensive research, its complex genetic mechanisms are still elusive, with multiple gene/loci currently identified and different modes of inheritance reported [28]. Our study found 7 differentially expressed proteins in tears of keratoconus patients, 4 of which are related to immune responses (immunoglobulin chains and neutrophil defensin). These results suggest the involvement of immunologic pathways in keratoconus pathophysiology. As the specific disease mechanisms in keratoconus are still obscure, any insight from its tear proteomic profile could aid future research.

Ocular surface disease is not a main feature in keratoconus patients. However, a previous study showed altered clinical parameters like tear break-up time (BUT), fluorescein and rose Bengal staining scores and lower corneal sensitivity [17]. Our study found the least number of altered proteins in the keratoconus group, which correlates to the lesser impact on the ocular surface in comparison to pterygium and GVHD related dry eye. It could also be related to the lower number of keratoconus subjects compared to the other study groups. Although the results from the Student’s t-test were not significant after multiple comparisons correction, the multivariate analyses were able to differentiate the keratoconus tear proteome from the control group through the heat map dendrogram analysis and the PCA scores plot. These tear proteome alterations in keratoconus could be directly related to increased cytokine secretion by the corneal epithelium or a concomitant ocular surface disease condition.

To our knowledge, we report the first findings of tear proteome in pterygium patients, comparing to a control group. Among the proteins with altered expression, Prolactin-inducible protein was previously reported as reduced in dry-eye patients [29, 30], while the protein S100A8 (calgranulin) was reported as increased in dry eye patients [31]. In our sample, we found increased expression of keratin proteins in pterygium and GVHD tears, which may be related to the increased epithelial keratinization that may happen in these conditions.

The estrogen signaling pathway was retrieved from the biochemical pathway prospection of the pterygium tear proteome. In a large cross-sectional population-based study with postmenopausal women [32], Kyung-Sun et al. found decreased pterygia prevalence among women receiving estrogen replacement therapy, in comparison to those not receiving estrogen replacement. They hypothesized that estrogen in the tear film might protect the ocular surface from pterygium development by blocking oxidative stress-induced inflammation. Although it is not yet possible to establish a causative effect, alterations in the estrogen signaling pathway could be related to pterygium pathophysiology and warrant further research.

The GVHD tear proteome showed the most altered profile of differentially expressed proteins, and several among them had already been described in previous studies. Protein S100-A9 is a proinflammatory protein with increased levels in tears from dry eye patients and positively correlated to disease intensity [29]. Immunoglobulin gamma-3 chain C was also found upregulated in tears from dry eye patients [30]. Histones are a group of DNA-binding proteins involved in nucleosome assembly and also described as pro-inflammatory mediators, previously reported in increased levels in tear samples from GVHD patients [11]. The proline-rich protein 4, found in decreased levels in tears from both GVHD and non-GHVD related dry eye, has been described as a product of the lacrimal gland, but its role on the ocular surface is not yet understood [6, 11, 31]. Lipocalin-1, a major component of normal tears, along with lysozyme C and lactotransferrin, both antimicrobial proteins, are produced by the lacrimal gland and are also downregulated in tears from dry eye patients [6, 11, 30]. Lacrimal gland dysfunction and fibrosis is a major feature of ocular GVHD [33], and it may explain the decreased level of the proteins discussed above. Interestingly, this downregulation of proteins with antibacterial activity like lysozyme and lactotransferrin may be related to the increased risk of infectious diseases of the ocular surface in dry eye patients [29].

The complement and coagulation cascades were retrieved by the biochemical pathway prospection from the GVHD tear protein profile. Previous studies have shown complement activation in GVHD patients, and also in transplant-associated thrombotic microangiopathy (TA-TMA), another complication of hematopoietic stem cell transplantation [34]. Endothelial injury would be the trigger to the complement activation in these conditions. Plasma complement component 3b (C3b) has also been identified in increased levels in TA-TMA and GVHD patients [35]. We have also found increased levels of complement component 3 (C3) in GVHD tears. These findings suggest that the tear film of GVHD patients may reflect the systemic alterations in complement cascade found in this disease.

There are several methods for tear sample collection, such as glass microcapillary tubes [3, 8, 10], Schirmer test I strips [6, 9, 30], and eye-flush with sterile saline or distilled water [36–38]. In this study, the eye flush method was chosen because of the technical difficulty in obtaining tear samples from the severe dry eye related GVHD group using either microcapillary tubes or Schirmer strips. Although the eye flush method may generate lower protein concentrations, it has been reported to yield the same spectrum of proteins in similar proportions as basal or reflex tear collection [38]. All study subjects had the same tear collection technique.

Proteomics experiments yield a large quantity of data, usually hundreds of different proteins. There is much debate in the literature on how to deal with all this information and which are the best statistical tools. Saccenti et al. [39], in a review article about the use of univariate and multivariate analysis of metabolomics data, suggest that both methods should be used, as they provide complementary information, and this is the strategy we used to analyze our data. We observed that in our sample some proteins appeared in the multivariate analysis, specifically the partial least squares discriminant analysis (PLS-DA), but they were not significant in the univariate analysis (Student t-test). These results do not necessarily match, and sometimes we can find significant results multivariately and not univariately. As multivariate methods use all variables simultaneously, we have information about the simultaneous relationship among them. Independent variables may complement each other and give information that is not always available through univariate methods. These results could be seen as complementary rather than contradictory.

In our study design, we intended to evaluate the tear film of three very distinct ocular diseases—keratoconus, pterygium, and chronic GVHD related dry eye, using LC–MS for proteomic investigation. Our purpose herein was to investigate in a pilot comparative study if ocular conditions with entirely different mechanisms and clinical presentations could be differentiated by tear proteome. Although our study had a small sample size, we could still demonstrate that each disease has a characteristic tear proteomic profiling, and the multivariate analysis, particularly PCA, was a powerful tool to differentiate the four study groups, showing the feasibility of the technique for future research with a larger sample size. The candidate biomarkers presented here are preliminary and need further validation. By understanding how these different conditions can modify the tear film proteome, these data may help future biomarker research and also provide insights into the pathophysiology of keratoconus, pterygium, and GVHD related dry eye. We hope this work will stimulate other research groups to increase the knowledge about the mechanisms involved in such broad areas of ocular disease.

## Conclusions

We demonstrated herein that mass spectrometry-based proteomics was able to indicate proteins that differentiate three distinct ocular conditions: keratoconus, pterygium, and GVHD related dry eye. We also reported potential candidates as biomarkers for each disease.

## Supplementary Information

**Additional file 1: Table S1.** Proteins identified in keratoconus and control group tears. Gene names, protein names and ID, and Student´s t-test results comparing all proteins identified in keratoconus and control group.

**Additional file 2: Table S2.** Proteins identified in pterygium and control group tears. Gene names, protein names and ID, and Student´s t-test results comparing all proteins identified in pterygium and control group.

**Additional file 3: Table S3.** Proteins identified in GVHD and control group tears. Gene names, protein names and ID, and Student´s t-test results comparing all proteins identified in GVHD and control group.

## Data Availability

All data generated or analyzed during this study are included in this published article and its additional files.
